# Differential expression of transcriptional regulatory units in the prefrontal cortex of patients with bipolar disorder: potential role of early growth response gene 3

**DOI:** 10.1038/tp.2016.78

**Published:** 2016-05-10

**Authors:** B Pfaffenseller, P V da Silva Magalhães, M A De Bastiani, M A A Castro, A L Gallitano, F Kapczinski, F Klamt

**Affiliations:** 1 Bipolar Disorder Program, Laboratory of Molecular Psychiatry, Hospital de Clínicas de Porto Alegre, Porto Alegre, Brazil; 2 Laboratory of Cellular Biochemistry, Department of Biochemistry, Universidade Federal do Rio Grande do Sul, Porto Alegre, Brazil; 3 Department of Psychiatry, Universidade Federal do Rio Grande do Sul, Porto Alegre, Brazil; 4 Bioinformatics and Systems Biology Laboratory, Federal University of Paraná, Polytechnic Center, Curitiba, Brazil; 5 Department of Basic Medical Sciences, University of Arizona College of Medicine, Phoenix, AZ, USA

## Abstract

Bipolar disorder (BD) is a severe mental illness with a strong genetic component. Despite its high degree of heritability, current genetic studies have failed to reveal individual loci of large effect size. In lieu of focusing on individual genes, we investigated regulatory units (regulons) in BD to identify candidate transcription factors (TFs) that regulate large groups of differentially expressed genes. Network-based approaches should elucidate the molecular pathways governing the pathophysiology of BD and reveal targets for potential therapeutic intervention. The data from a large-scale microarray study was used to reconstruct the transcriptional associations in the human prefrontal cortex, and results from two independent microarray data sets to obtain BD gene signatures. The regulatory network was derived by mapping the significant interactions between known TFs and all potential targets. Five regulons were identified in both transcriptional network models: early growth response 3 (*EGR3*), TSC22 domain family, member 4 (*TSC22D4*), interleukin enhancer-binding factor 2 (*ILF2*), Y-box binding protein 1 (*YBX1*) and MAP-kinase-activating death domain (*MADD*). With a high stringency threshold, the consensus across tests was achieved only for the *EGR3* regulon. We identified *EGR3* in the prefrontal cortex as a potential key target, robustly repressed in both BD signatures. Considering that *EGR3* translates environmental stimuli into long-term changes in the brain, disruption in biological pathways involving *EGR3* may induce an impaired response to stress and influence on risk for psychiatric disorders, particularly BD.

## Introduction

Bipolar disorder (BD) is a severe mental illness with a strong genetic component. Heritability is high, as evaluated by monozygotic and dizygotic twin concordance, albeit not perfect.^1, 2^ Nevertheless, current molecular genetic studies indicate that no particular locus of large effect is involved in its etiology. The candidate gene approach has not delivered robust results and genome-wide association studies also often fail to show strong positive signals.^2^ Current polygenic analyses are consistent with hundreds or thousands of susceptibility variants of weak effect, with the variation in single-nucleotide polymorphisms explaining 20 to 30% of the heritability shown in family and twin studies.^3, 4^

The generally low yield of pure genetic association studies has generated interest in alternative approaches.^5^ Gene expression studies move the discussion beyond statistical associations into the realm of neurobiology. RNA analysis can be more informative of the status of the cell, as it reflects a functional state not only influenced by genetic polymorphisms, but also by transcriptional modulation. Using high-throughput technologies, such as microarrays, the differential expression of genomic DNA in the form of mRNA has the potential to lead to disease signatures.^6^ One major caveat is that gene expression is tissue specific. For BD studies, this means obtaining brain tissue from postmortem donors, and few brain bank collections exist for this illness.^7^

Given the difficulty of obtaining brain tissue, the data on gene expression in BD is quite limited (compared with cancer research, for example). To get an idea of the sparseness of existing data in BD, a recent systematic review of gene expression studies revealed publicly available data from only 57 unique BD cases.^8^ Furthermore, gene expression varies depending on the area and structure under study.^9^ This underscores the importance of selecting the appropriate brain regions and using a methodological framework to extract biologically meaningful information from large-scale data. Although the neurocircuitry involved in the mood disorders is expected to be complex, there are major areas of interest that could be fruitfully explored in postmortem studies. Overall, interest has focused on the limbic–cortical–striato–pallido–thalamic circuits. The prefrontal cortex is a relevant nexus, where recent research points to areas in the medial prefrontal and orbitofrontal cortex.^10^

In the field of systems biology, approaches to identify candidate master regulators (MRs) have focused on transcription factors (TFs) that exert large influences on a phenotype. Recently, a number of computational methods have been developed to identify groups of genes, and even entire pathways, coordinated by a small number of TFs. These approaches have successfully identified gene units that are impaired in diseases such as cancer and diabetes.^11, 12, 13, 14, 15^ As in other traditional medical fields, psychiatry is currently focusing on the study of biological pathways. Reverse engineering algorithms are used to reconstruct cell type-specific regulatory networks from high-throughput data. This approach efficiently reduces the complexity of the network, allowing the identification of MR TFs with tissue-specific signatures.

Here we query the genetic regulatory signature of BD through a series of steps. First, we analyze a large gene expression data set from healthy human prefrontal cortex across the lifespan^16^ to construct a regulatory network of known TFs and all potential targets. As gene expression is tissue specific, this empirical approach has the benefit of being a more realistic representation of prefrontal cortex functioning. Next, we identify genes that are differentially expressed in the prefrontal cortex of patients and healthy controls from two separate data sets of postmortem tissue samples. We reasoned that if the BD gene expression signature in the prefrontal cortical regulatory units is influenced by the activation (or repression) of specific TFs, then both the downstream targets, as well as the upstream regulators, of these TFs should be among the most differentially expressed genes in the BD phenotype. The use of network-based approaches to elucidate biological mechanisms of complex diseases may allow a clearer view of the molecular networks governing the pathophysiology and reveal potential targets for drug design and therapeutic intervention.

## Materials and methods

### Microarray data

The data used to reconstruct the transcriptional associations in the human prefrontal cortex was obtained from a large-scale microarray study describing an extensive series (*n*=269) of brain tissue samples from fetal development through aging,^16^ downloaded from GEO (accession number GSE30272). Two studies using independent microarray platforms (Affymetrix Human Genome 133A GeneChips and Codelink Expression Assay arrays) were used to obtain the BD gene expression signatures (accession numbers GSE12679 and GSE5388). The two selected data sets were the best and most interesting sets available to our goal. As we aimed to evaluate differential expression of transcriptional regulatory units in the prefrontal cortex of bipolar disorder patients, we sought the most specific samples available (data set 1—data from pyramidal neurons) and other presenting more sensibility (data set 2—data from all cortical cells), both data sets from the same prefrontal area (Brodmann area 9—BA9). As such, the samples and raw data were independently processed and generated, contributing to check the consistency of the regulatory units characterized in our study.

For the first data set, the samples were obtained using laser-capture microdissection^17^ and included 1000 pyramidal neurons isolated from region BA9 of prefrontal cortex from five individuals with BD and six control subjects. For the second data set, fresh-frozen prefrontal cortex tissue (region BA9) was obtained from the Neuropathology Consortium of the Stanley brain collection (Stanley Medical Research Institute, Chevy Chase, MD, USA)^18^ and included 30 BD and 31 control samples. The demographic variables for the samples have been scrutinized in the original studies that produced the public data. For the first data set, the information available includes age (years): patients—43.4±6.3, controls—41.2±7.2; gender (F/M): patients—1/4, controls—2/4; postmortem interval (hours): patients—28.2±5.4, controls—30±18.6. The study related to the second data set presents more detailed information that is summarized below. Age (years): patients—44.5±10.7, controls—43.8±7.3; gender (F/M): patients—14/16, controls—7/24; brain pH: patients—6.48±0.27, controls—6.62±0.27; postmortem interval (hours): patients—37.2±17.7, controls—29.1±13.1; suicide (Y/N): patients—12/18, controls—0/31; alcohol abuse (none or light/moderate to severe): patients—13/17, controls—27/4. Regarding treatment of patients, lithium (Y/N): 8/22; valproate (Y/N): 10/20; electroconvulsive therapy history (Y/N): 2/28. Some potential confounding issues are not available to be controlled; however, they are addressed in the discussion.

### Transcriptional network inference

The transcriptional networks were constructed using the R package RTN.^19^ Regarding the code availability used in this study, it is publicly available from Bioconductor in the R packages RTN (http://bioconductor.org/packages/RTN/). Gene probes (complementary sequences to the target mRNAs used in microarray to assay gene expression) were filtered based on their coefficient of variation and mutual information was calculated in the R package *minet*.^20^ The regulatory structure of the network is derived by mapping the significant interactions between known TFs and all potential targets in the gene expression matrix. The interactions that are below a minimum mutual information threshold are eliminated by permutation analysis. Unstable interactions are additionally removed by bootstrap analysis using 1000 bootstrap samples to create the consensus bootstrap network (that is, the relevance network). In an additional step, the Data Processing Inequality (DPI) algorithm is applied with tolerance=0.0 to eliminate interactions that are likely to be mediated by another TF.^11^ As the DPI removes the weakest edge of each network triplet, the vast majority of the interactions that are likely to be indirect are eliminated in this step. The resulting DPI-filtered transcriptional network is subsequently interrogated in the enrichment analysis. Both DPI-filtered and unfiltered transcriptional networks are used to visualize the final results. The analysis pipeline, resampling procedures and methods used to reconstruct the transcriptional networks are summarized in Supplementary Figure 1.

### MR and gene set enrichment analysis

The MR analysis is described elsewhere.^15^ Briefly, the gene set enrichment analysis (GSEA) is used to assess whether a given transcriptional regulatory unit (regulon) is enriched for genes that are differentially expressed among two classes of microarrays. The GSEA uses a rank-based scoring metric obtained from the differentially expressed signatures to test the association between gene sets and the ranked phenotypic difference. The current analysis treats regulons as gene sets, and the BD signatures as the phenotype, in an extension of the GSEA analysis as previously described.^21^ The GSEA was performed in the R package RTN using 1000 permutations.

### Two-tailed GSEA

The two-tailed GSEA assesses the direction of inferred connection between a given MR and the differentially expressed signatures, a proxy for induced or repressed associations. The method is based on the Connectivity Map procedure.^22^ The regulon is split into two subgroups, positive targets (*A*) and negative targets (*B*) using Pearson's correlation, whereas genes in the phenotype are ranked using the differentially expressed signatures (that is, top-down phenotype). The distribution of *A* and *B* are then tested by the GSEA statistics in the ranked phenotype, producing independent enrichment scores (ES) for each subgroup. A good separation of the two distributions and maximum deviation from zero near opposite extremes is desirable for a clear association. Therefore, an additional step is executed testing the differential enrichment (ES*_A_*−ES*_B_*). A high positive differential score indicates that the phenotype induced the regulon, whereas a high negative differential score indicates that the phenotype repressed the regulon. The two-tailed GSEA was performed in R using the function *tni.gsea2* in the RTN package with 1000 permutations.

### Analysis of gene expression data

The Bioconductor package *limma*^23^ was used to call differentially expressed genes, and the log fold change (logFC) metric was used to obtain the ranked phenotypes required for the GSEA analysis.

## Results

### A tissue-specific regulatory network for the human prefrontal cortex

**Figure 1:**
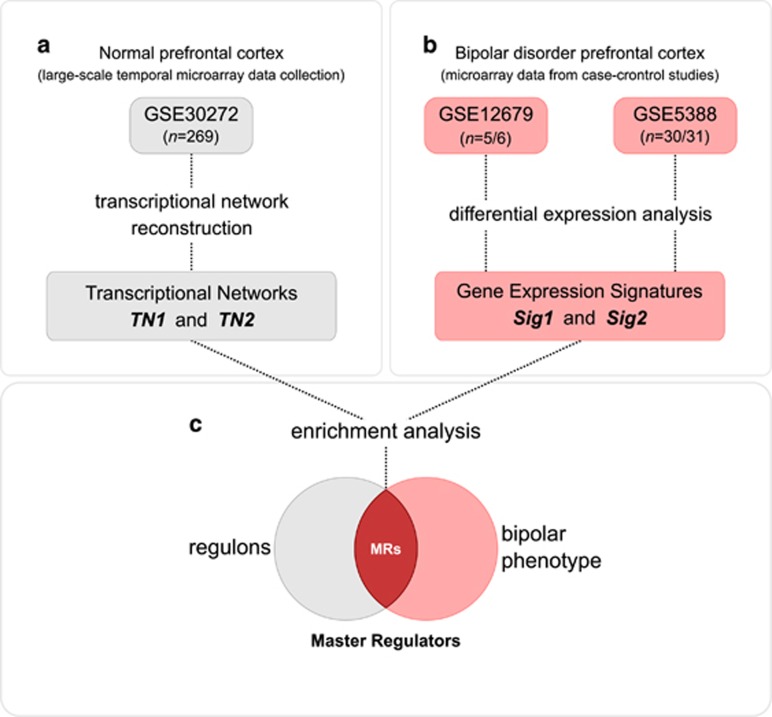
Master regulator (MR) analysis flowchart. (**a**) Data source used to reconstruct the transcriptional regulatory units in normal human prefrontal cortex. A large-scale microarray study (GSE30272) describing an extensive series of brain tissue from fetal development through aging was used to compute the transcription factor-centric regulatory networks (regulons). The transcriptional network *TN1* represents the totality of the 269-microarray samples in the study, whereas the *TN2* derives from a subsample with adult human prefrontal cortex only. The analysis pipeline, resampling procedures and methods used to reconstruct the transcriptional networks are further detailed in the Supplementary Figure 1. (**b**) Flowchart summarizing the microarray data used to obtain two independent bipolar disorder gene expression signatures (bipolar phenotypes). Signature 1 (*Sig1*; GSE12679) is derived from laser-capture microdissected human neurons isolated from postmortem dorsolateral prefrontal cortex, whereas signature 2 (*Sig2*; GSE5388) is derived from human postmortem brain tissue from adult subjects. (**c**) The enrichment analysis aims to identify transcriptional regulatory units associated with the gene expression signatures.

**Figure 2:**
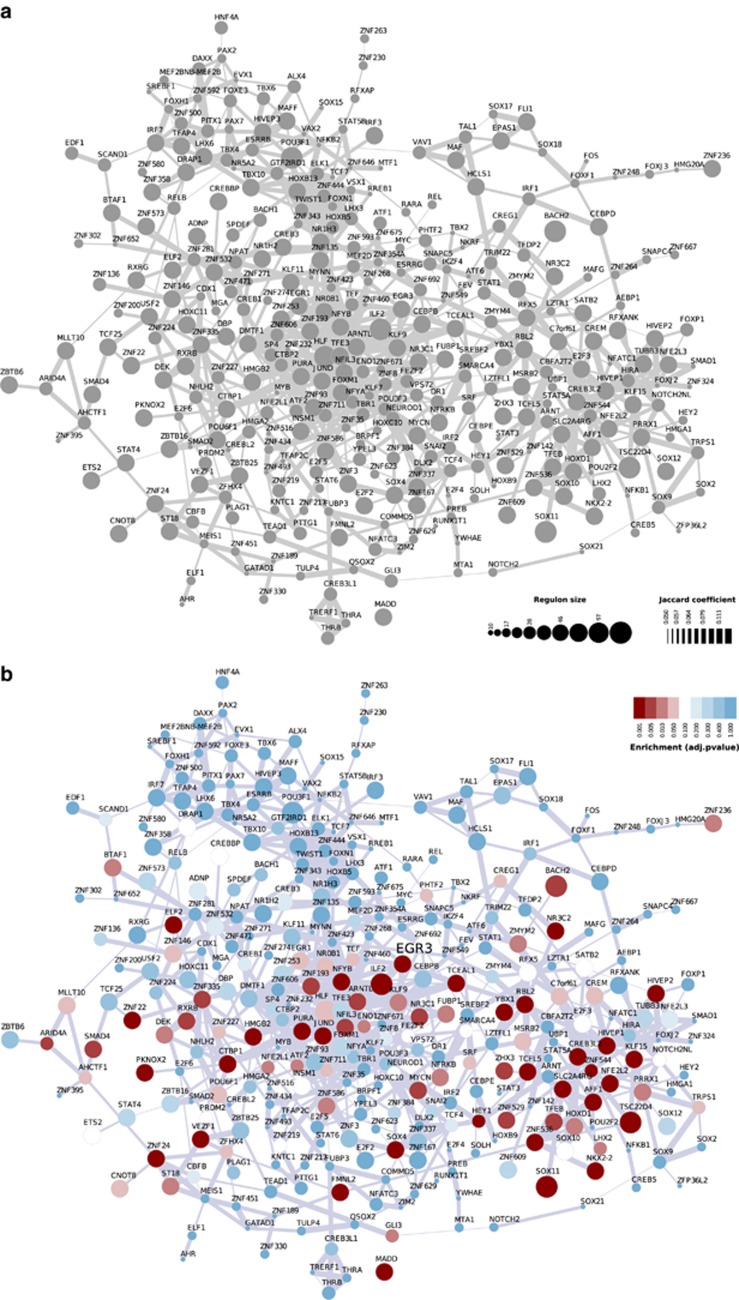
A systems model of the human prefrontal transcriptional network. (**a**) Association map showing the degree of similarity among regulons in the transcriptional network *TN1*. The node size represents the number of transcription factor (TF)–targets in the relevance network, whereas edge width corresponds to the overlap between regulons assessed by the Jaccard coefficient (JC). Unconnected regulons are not shown. (**b**) Enrichment analysis using gene expression signature 1 (*Sig1*) showing the distribution of the bipolar phenotype onto the association map (adjusted *P*-value <0.05 are shown in red-color scale).

We first established a tissue-specific transcriptional network model computed from a large-scale human prefrontal microarray data set (transcriptional network reconstruction summarized in Figure 1a). The microarray data were pre-processed and probes with low variation were removed from the analysis. Two transcriptional networks (*TN1* and *TN2*) were then derived by computing the mutual information between annotated TFs and all potential targets in the data set. *TN1* represents the totality of the 269-microarray samples in the study, whereas *TN2* is derived from a subsample with adult human prefrontal cortex only (see Supplementary Figure 1 for additional details of the resampling procedures). The association map in Figure 2a summarizes the transcriptional network *TN1* and shows the degree of similarity among the inferred regulatory units (regulons). The node size represents the number of targets in a given regulon, whereas edge width corresponds to the number of common targets between any two regulons assessed by the Jaccard coefficient (JC). In this reference network, each target can be linked to multiple TFs and regulation can occur as a result of both direct (TF–target) and indirect interactions (TF–TF–target). To preserve the dominant TF–target pairs for the subsequent enrichment analyses, we additionally applied the DPI algorithm, which removes the weakest interaction in any eventual triplet formed by two TFs and a common target gene (see methods and computational pipeline summarized in Supplementary Figure 1).

### MRs in BD prefrontal cortex

The inferred transcriptional network model was next used to query regulons enriched for the BD gene expression signatures. These signatures were obtained by differential expression analysis using microarray data from the two independent case–control sets (Figure 1b). Signature 1 (*Sig1*) is derived from laser-capture microdissected human neurons isolated from postmortem dorsolateral prefrontal cortex, whereas signature 2 (*Sig2*) is derived from human postmortem brain tissue from adult subjects. The MR analysis^13^ aims to identify regulons associated with the gene expression signatures (Figure 1c). Therefore, our primary goal here is to generate hypotheses regarding the transcriptional regulation in BD, identifying the MRs responsible for coordinating the activity of the signature genes. We used the GSEA statistics to test the enrichment of the signature genes in each regulon. Figure 2b presents the results of the GSEA analysis using *TN1* and *Sig1* and shows the distribution of the BD phenotype onto the transcriptional association map.

**Figure 3:**
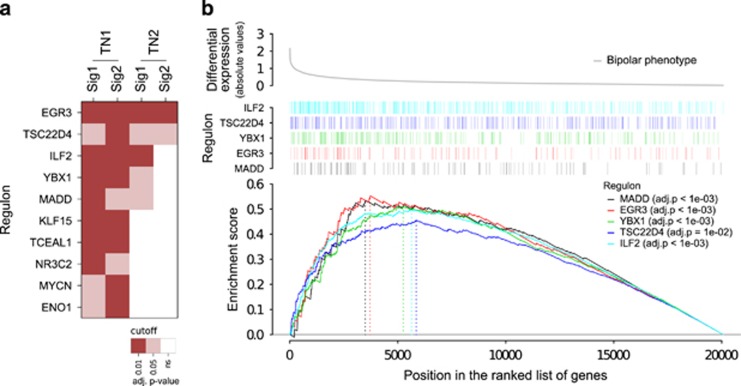
Consensus master regulators enriched for the bipolar disorder signatures. (**a**) Gene set enrichment statistics showing the regulatory units consistently enriched for the expression signatures *Sig1* and *Sig2* on the transcriptional network *TN1*, together with the results obtained for the same regulons on *TN2*. (**b**) Gene set enrichment plots showing the distribution of the top-five master regulators (that is, the consensus regulatory units) across the ranked bipolar phenotype represented by the absolute differential expression values (absolute logFC) derived from *Sig1*. The enrichment score is obtained based on the distribution of the hits: the *x* axis indicates the position of all genes ranked by the phenotype, and the hits indicate the position of each gene of a given regulon (see methods for additional description on the GSEA statistics). ns, not significant.

Among the several candidates identified, 10 regulons were significantly enriched for both gene expression signatures (Figure 3a), five of which are consensus in both *TN1* and *TN2* transcriptional network models: early growth response protein 3 (*EGR3*), TSC22 domain family, member 4 (*TSC22D4*), interleukin enhancer-binding factor 2 (*ILF2*), Y-box binding protein 1 (*YBX1*) and MAP-kinase-activating death domain (*MADD*). When a high stringent threshold is applied, the overall consensus across all the tests is only obtained for the regulon of the *EGR3*. The GSEA plots in Figure 3b shows the distribution of the top-five consensus MRs in the BD phenotype.

### Mode of action of the computationally defined regulons

**Figure 4:**
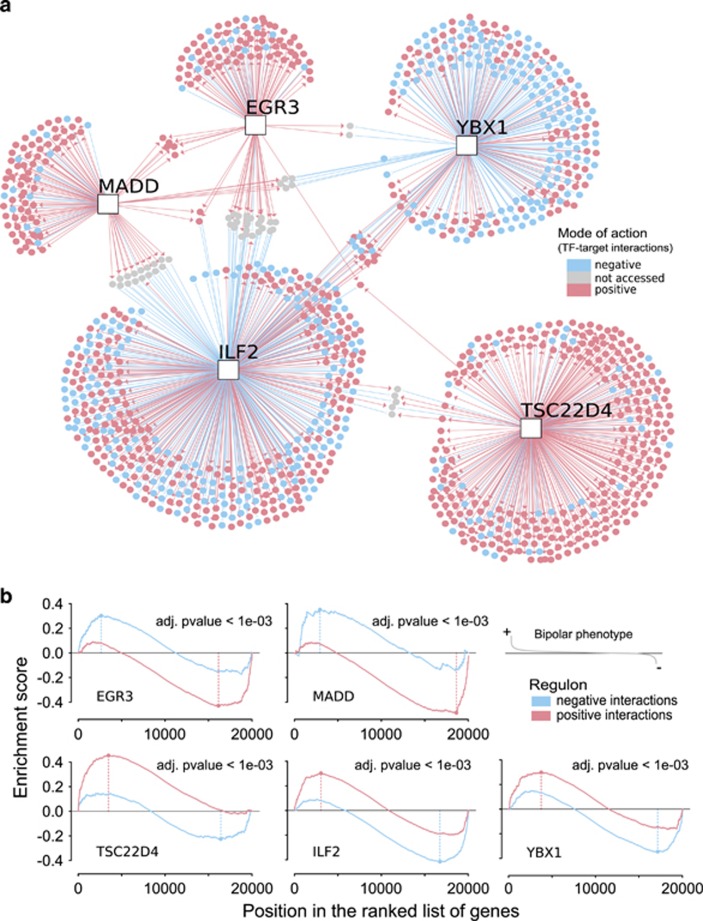
Regulatory units associated with the bipolar disorder phenotype. (**a**) The regulatory network shows the transcription factor (TF)–target interactions of the five master regulators, each one comprising one TF (square nodes) and all inferred targets (round nodes). The mode of action represented in red/blue colors corresponds to the correlation pattern observed between a given transcription factor and its targets, assessed by the Pearson's correlation on *TN1*. (**b**) Two-tailed gene set enrichment analysis. The enrichment plots show the distribution of the genes in each regulon across the ranked phenotype derived from *Sig1*. Regulons are split in positive (red) and negative (blue) targets, whereas the phenotype is ranked from the highest (+) to the lowest (−) differential expression values (logFC), that is, from the most increased to the most decreased gene expression values.

To visualize the five MRs, we show in Figure 4a the correlation pattern observed between the TFs (square nodes) and its inferred targets (round nodes) assessed by the Pearson's correlation on *TN1*. This network graph shows all interactions inferred for each regulon, whether positive (red targets) or negative (blue targets). Using this information, we extended the gene set enrichment analysis in order to access how the mode of action of these regulons are connected with the BD gene signatures. We used a two-tailed GSEA statistics on regulons split into positive (red) and negative (blue) targets (Figure 4b), and the resulting distributions were tested against the BD phenotype ranked from the highest (+) to the lowest (−) differential expression values.

Accordingly, *EGR3* and *MADD* negative targets are associated with the positive phenotype (that is, most induced genes), whereas the positive targets are associated with negative phenotype (that is, most repressed genes), providing a high negative differential score with adjusted *P*-value <0.001. It suggests that both *EGR3* and *MADD* regulons are repressed in the BD gene signature, whereas the other three regulons appear to be increased.

## Discussion

The primary goal of this study was to generate hypotheses regarding transcriptional regulation in BD, and to identify putative regulatory units that are dysfunctional in the prefrontal cortex of patients. To that end, we sought differentially expressed signatures that converged from two available gene expression data sets with distinctive strengths and weaknesses. Using MR analysis, our major finding was that the *EGR3* regulon was robustly repressed in both BD gene expression signatures. Four additional MRs showed a lower level of association with the BD phenotype.

*EGR3* is a member of the *EGR* gene family of immediate early genes transcription factors. These genes are expressed at basal levels throughout the brain, including the cortex, hippocampus and other limbic areas, and the basal ganglia.^24^ *EGR* expression is induced at high levels in response to environmental events and stressful stimuli across a range of intensities. In the brain, this activation is triggered by neurotransmitter-receptor stimulation or depolarization.^24^ Numerous behavioral and electrophysiologic studies in animals have shown that the *EGR* family has a role in memory acquisition and consolidation and hippocampal synaptic plasticity.^24, 25, 26, 27, 28, 29^ *EGR3*, in particular, is required for the normal response to stress as well as in the neuroplasticity induced by this responsivity, ultimately regulating neuronal gene expression.^27, 30, 31^

Of particular relevance are studies demonstrating that mice lacking functional *EGR3* display behavioral and physiologic abnormalities consistent with models of mental illness. These include a heightened response to stress (evidenced behaviorally and by elevated release of corticosterone), hyperactivity and failure to habituate to environmental stimuli and social cues.^27^ The hyperactivity, a rodent psychosis phenotype, is reversible with antipsychotic medications used to treat BD.^32, 33^ *EGR3* regulates expression of important plasticity associated genes, such as those encoding the activity regulated cytoskeletal associated gene (*Arc*)^30, 34^ and GABA receptor subunit 4 (*GABRA4*),^35^ and other member of the *EGR* family regulates the synaptic vesicle associated proteins *synapsin 1*^36^ and *synapsin 2*.^37^ Thus, requirement of *EGR3* in processes of memory, learning and synaptic plasticity is likely to be mediated by these, and presumably other as-yet unidentified, target effector genes.

The neuronal expression of *EGR3* is regulated by synaptic activity and is coupled to MAPK-ERK signaling.^24, 28^ Gallitano-Mendel and colleagues noted that *EGR3* is activated downstream of numerous proteins associated with risk for psychotic illness, including neuregulin 1 (*NRG1*), calcineurin (*CN*) and *N*-methyl-d-aspartate (*NMDA*) receptors.^27, 32, 38, 39, 40, 41^ Moreover, drugs that induce psychosis via serotonin 2A receptors (5-HT2ARs) regulate expression of *EGR3*.^42^ They have hypothesized that these genes, together with targets of *EGR3*, comprise a pathway of proteins which, when disrupted at any level, increases risk for psychotic illness. In addition, brain-derived neurotrophic factor (*BDNF*) has been shown to induce *EGR3* expression via a PKC/MAPK-dependent pathway.^35^ These are all interesting links, as *BDNF* has been proposed as a critical factor in the reduced cellular resilience associated to BD.^43, 44^ A growing body of data has shown that peripheral *BDNF* levels are decreased during BD episodes and with the illness progression.^45, 46^ Despite limitations in the studies and conflicting results in this area, it is intriguing to speculate that reduced peripheral *BDNF* levels, whether related to its decreased expression in the brain, may influence the *EGR3* repression as this TF is regulated by *BDNF*. Obviously, further research is warranted for substantially improving the knowledge regarding the link between *BDNF* and *EGR3* in a shared biological pathway and their role in BD.

Downstream, *EGR3* targets the promoter region of genes involved in neuroplasticity or stimuli response. So far, experimental studies show effects on NMDA receptor subunits NR1 and NR2B, and type A GABA receptor, and possibly on genes involved in microglia deregulation associated with psychiatric disorders, such as the triggering receptor expressed on myeloid cells 1 (*TREM-1*).^27, 35, 47, 48^ *EGR3* also regulates the expression of NGFR (*p75NTR*),^49^ a receptor for neurotrophins that is involved in the regulation of axonal elongation.^50^ Perhaps most intriguing is the recent findings that the *EGR3* target gene *ARC*, which modifies synapses in response to environmental stimuli,^51^ is implicated in risk for psychotic disorders.^41, 52, 53, 54^ Altogether, *EGR3* targets trigger different downstream genes and pathways involved in processes such as synaptic plasticity, axon extension, regulation of *BDNF* and receptors expression, among others.^55^

EGRs translate environmental events into long-term changes in neural gene expression. This has led to the hypothesis that dysfunction in EGRs may account for both the genetic and environmental influences on risk for psychiatric illnesses.^32, 41, 56^ The *EGR* family has been more closely scrutinized in schizophrenia, with fewer and less-consistent studies in BD.^47, 56, 57, 58^ *EGR3* has been significantly associated with schizophrenia in the Japanese,^40^ Korean and Han Chinese populations,^59, 60^ and recently in a population of European descent.^41^ Although not all studies have found significant associations,^57, 61, 62^ a meta-analysis of the studies in Asian populations supported association between *EGR3* and schizophrenia.^60^ In addition, the AA genotype of the *rs35201266* SNP was recently associated with the hemodynamic state of the prefrontal cortex in both patients with schizophrenia and healthy participants, possibly suggesting a pathway from neurodevelopment to brain function.^63^ Furthermore, a study involving an entire network of TFs and microRNAs related to schizophrenia identified *EGR3* as the central gene in the regulatory network.^55^

Studies examining a potential role for *EGR3* in BD identified nominal associations that did not meet the threshold for significance following the strict Bonferroni correction for multiple comparisons. In the first study, examining association of genes involved in circadian rhythms with BD, *EGR3* was to sole gene that achieved a significance level of *P*<0.05.^57^ A second investigation, a family-based association study identified a nominal association of *EGR3* with risk for child with BD I.^56^ These findings suggest that *EGR3* may be a fruitful gene for future genetics studies to identify mechanisms by which environment and genetic predisposition interact to influence BD. Although the statistically significant findings supporting an association between *EGR3* and psychiatric illness have been in schizophrenia, research has increasingly demonstrated that the molecular and genetic processes underlying BD and schizophrenia are highly coincident.^64^

The possible effects of mood stabilizers, psychotropic medications and substance use on the *EGR3* regulon are also an interesting point to be discussed. However, there are few studies on this matter, most of them evaluating other *EGR* genes. To our knowledge, there are no studies showing association between lithium or valproate effects and *EGR3* expression. In this context, studies observed that the expression of *EGR1* was increased by lithium in mouse frontal cortex,^65^ and by valproate in neural stem cells.^66^ Considering that lithium has been associated with neurogenesis, it is conceivable that it induces *EGR* genes as well. Nevertheless, our results point to the repression of this regulon in bipolar disorder, suggesting that lithium treatment did not influence our findings. Studies in rodents have shown that other psychoactive medications induce immediate early genes in the brain. For instance, chronic treatment with aripiprazole induces differential gene expression of *EGR1*, *EGR2* and *EGR4* in the rat frontal cortex;^67^ *EGR1* is differentially expressed also in rat striatum after haloperidol and clozapine treatments.^68^ Though less studied than *EGR1*, expression of *EGR3* is induced by several of the same stimuli of *EGR1*, including antipsychotic medications or drugs that induce psychosis.^25, 42^ Other factors that might possibly affect the *EGR3* regulon is alcohol or substance use; a relationship between drug intake or withdrawal and induction of *EGR* genes has been reported. For instance, amphetamine and cocaine increase *EGR1* mRNA expression in the striatum,^69^ cocaine also induce *EGR3* in the striatum^25^ and amphetamine or alcohol withdrawal induce *EGR1* expression.^70^ Considering these observations, it seems unlikely that antipsychotic treatment, alcohol or other substances are responsible for our findings, as they induce *EGR3* and other growth response genes, whereas our results pointed to the repression of the *EGR3* regulon in BD signatures.

Our data suggest that decreased function of *EGR3* may be involved in BD. As a MR of a network of genes and pathways that mediate critical neurobiological processes, dysfunction in *EGR3* indicates a possible explanation for both the influence of environment, as well as the role of numerous genes in the pathogenesis of BD. The identified network thus provides potential targets for follow-up experimental evaluation and development of novel therapeutics for this severe mental illness. The results presented here are both innovative and exploratory, and are therefore in need of confirmation before more definitive assertions regarding the relevance of *EGR3* in BD can be made. A new generation of bioinformatics methods has been developed to deal with the notorious limitations of functional genomics data.^12^ Nevertheless, further validation through basic science laboratory approaches, including mRNA expression of *EGR3* and key interacting genes in BD postmortem PFC tissue using PCR with reverse transcription, is an important step towards firmly confirming our results. However, studies in *EGR3*-deficient mice demonstrating psychosis-like phenotypes and hyperactivity that can be reversed with antipsychotic medications that are used in treatment of BD already provide important support for our findings.^27, 32, 33^

Limitations of this study include the fact that only two microarray sets were used to obtain the signatures. Although we do not intend to perform an exhaustive analysis of all regions and all available data sets, our bioinformatics approach is constrained by the availability of a unique cohort study with a large sample size (to compute the regulatory unities) and the gene expression signatures interrogating the tissue under study. The starting point for our analysis was the public availability of a unique study, which sampled prefrontal cortex from people with bipolar disorder obtained using laser-capture microdissection. As this study was limited by sample size, and there are no other analyses using this technique, we next sought the largest available data set that used brain homogenates from the same prefrontal area (BA9), and we found just one study with these criteria. Hence the two data sets were formed not by all sets, but by the best and most interesting sets available. Other limitation is that the analysis was restricted to prefrontal cortex (BA9) and gene expression profiles might look different in other laminar and brain regions. Future studies should aim at evaluating laminar and regional specificity of our results, and validating our findings with biochemical/molecular analyses in independent biological samples, as well as studying *EGR3* targets, their role in BD and in the mechanisms of action of drugs. When it comes to the means of modulation of *EGR3*, it is likely that genetic and epigenetic mechanisms might be underlying the alterations seen in BD, which warrants further studies on this matter as well.

In conclusion, we have used an innovative approach based on MR analysis to study transcriptional regulation in BD. This method identified the *EGR3* gene as a potential key target, with the *EGR3* regulon robustly repressed in both of the two BD gene expression data sources we examined from postmortem prefrontal cortex. Considering that *EGR3* is activated throughout the brain in response to stressful environmental stimuli, the possible disruption in biological pathways involving *EGR3* may result in an impaired response and adaptation to stress. This could result in reduced neurobiological resilience and ultimately lead to the symptoms of executive and cognitive dysfunction seen in BD. The bioinformatics approach used in this work may give insights for identifying targets possibly involved with the risk for psychiatric disorders and inspire drug-discovery programs that can affect these disorders.

## Notes

Professor Kapczinski has received grant/research support from Astra-Zeneca, Eli Lilly, the Janssen-Cilag, Servier, CNPq, CAPES, NARSAD and the Stanley Medical Research Institute; has been a member of the speakers' boards for Astra-Zeneca, Eli Lilly, Janssen and Servier; and has served as a consultant for Servier. The remaining authors declare no conflict of interest.
