# Supplementary figures and images for: Differential expression of transcriptional regulatory units in the prefrontal cortex of patients with bipolar disorder: potential role of early growth response gene 3
Source: Transl Psychiatry. 2016 May 10;6(5):e805–. doi: 10.1038/tp.2016.78 (PMC5070056; doi:10.1038/tp.2016.78)

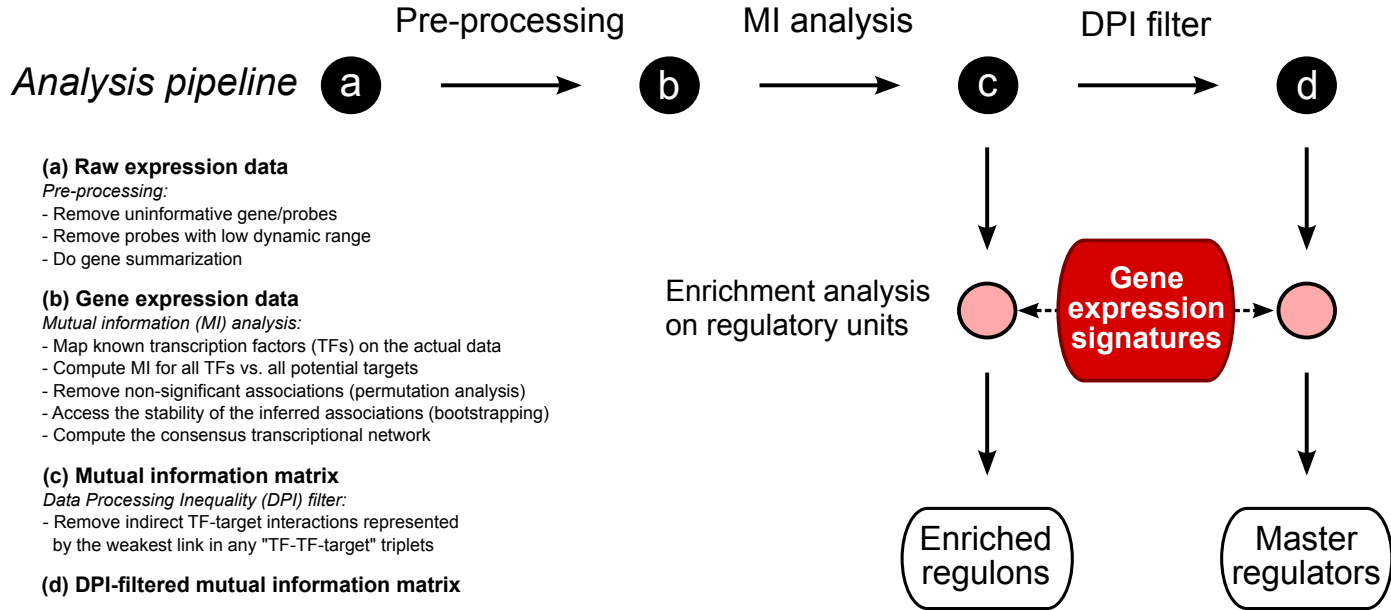

Supplement: Supplementary Figure 1 [file tp201678x1.pdf]
