# Supplementary material for: Differential expression of transcriptional regulatory units in the prefrontal cortex of patients with bipolar disorder: potential role of early growth response gene 3
Source: Transl Psychiatry. 2016 May 10;6(5):e805–. doi: 10.1038/tp.2016.78 (PMC5070056; doi:10.1038/tp.2016.78)
Supplement: Supplementary Figure Legend [file tp201678x2.doc]

**Supplementary Figure 1**. **Network inference and master regulator analysis flowchart.** The gene expression datasets were analyzed with the ARACNe algorithm (11) to derive the TF-centric regulatory networks. Master regulators were identified by examining the enrichment of the targets in each regulon for the gene expression signatures using the gene set enrichment analysis (GSEA). The reconstruction of the transcriptional networks was executed in the *R* package *RTN* (19) and all intermediate steps listed in the analysis pipeline were carried out in *R*.
